# Mitochondrial Transplantation’s Role in Rodent Skeletal Muscle Bioenergetics: Recharging the Engine of Aging

**DOI:** 10.3390/biom14040493

**Published:** 2024-04-18

**Authors:** Tasnim Arroum, Gerald A. Hish, Kyle J. Burghardt, James D. McCully, Maik Hüttemann, Moh H. Malek

**Affiliations:** 1 Center for Molecular Medicine and Genetics, Wayne State University, Detroit, MI 48201, USA; ho0066@wayne.edu (T.A.); ah6179@wayne.edu (M.H.); 2 Unit for Laboratory Animal Medicine (ULAM), University of Michigan, Ann Arbor, MI 48109, USA; 3 Department of Pharmacy Practice, Eugene Applebaum College of Pharmacy and Health Sciences, Wayne State University, Detroit, MI 48201, USA; 4 Department of Cardiac Surgery, Boston Children’s Hospital, Harvard Medical School, Boston, MA 02115, USA; 5 Physical Therapy Program, Department of Health Care Sciences, Eugene Applebaum College of Pharmacy and Health Sciences, Wayne State University, Detroit, MI 48201, USA; 6 Integrative Physiology of Exercise Laboratory, Department of Health Care Sciences, Eugene Applebaum College of Pharmacy and Health Sciences, Wayne State University, Detroit, MI 48201, USA

**Keywords:** exercise physiology, endurance, energy production

## Abstract

Background: Mitochondria are the ‘powerhouses of cells’ and progressive mitochondrial dysfunction is a hallmark of aging in skeletal muscle. Although different forms of exercise modality appear to be beneficial to attenuate aging-induced mitochondrial dysfunction, it presupposes that the individual has a requisite level of mobility. Moreover, non-exercise alternatives (i.e., nutraceuticals or pharmacological agents) to improve skeletal muscle bioenergetics require time to be effective in the target tissue and have another limitation in that they act systemically and not locally where needed. Mitochondrial transplantation represents a novel directed therapy designed to enhance energy production of tissues impacted by defective mitochondria. To date, no studies have used mitochondrial transplantation as an intervention to attenuate aging-induced skeletal muscle mitochondrial dysfunction. The purpose of this investigation, therefore, was to determine whether mitochondrial transplantation can enhance skeletal muscle bioenergetics in an aging rodent model. We hypothesized that mitochondrial transplantation would result in sustained skeletal muscle bioenergetics leading to improved functional capacity. Methods: Fifteen female mice (24 months old) were randomized into two groups (placebo or mitochondrial transplantation). Isolated mitochondria from a donor mouse of the same sex and age were transplanted into the hindlimb muscles of recipient mice (quadriceps femoris, tibialis anterior, and gastrocnemius complex). Results: The results indicated significant increases (ranging between ~36% and ~65%) in basal cytochrome *c* oxidase and citrate synthase activity as well as ATP levels in mice receiving mitochondrial transplantation relative to the placebo. Moreover, there were significant increases (approx. two-fold) in protein expression of mitochondrial markers in both glycolytic and oxidative muscles. These enhancements in the muscle translated to significant improvements in exercise tolerance. Conclusions: This study provides initial evidence showing how mitochondrial transplantation can promote skeletal muscle bioenergetics in an aging rodent model.

## 1. Introduction

Cardiorespiratory fitness is a health indicator of all-cause mortality [1,2]. One critical component of cardiorespiratory fitness is the function of mitochondria within the skeletal muscle which generates energy to perform exercise or activities of daily living. There is clear evidence that aging results in a reduction in mitochondrial function [3,4]. Initially proposed by Harman as the mitochondrial theory of aging in the 1950s [5], age-related decreases in mitochondrial function have since been shown to play a major role in skeletal muscle decline. Not surprisingly, in regard to aging-related decline of skeletal muscle, mitochondrial oxidative capacity has been implicated in sarcopenia [6,7,8,9]. Research suggests that the skeletal muscle of elderly individuals exhibits a rise in nonoperational mitochondria, an increase in mutated and deleted mitochondrial DNA with an associated decrease in mitochondrial density [10]. Mitochondria do not only generate more than 90% of the cells’ energy, but they are also the main source of reactive oxygen species (ROS), causing oxidative stress and accumulation of cellular damage over time. This in turn leads to redox homeostasis imbalance, mitochondrial dysfunction, and ultimately cellular demise. 

As the proposed rate-limiting enzyme in the electron transport chain (ETC), cytochrome *c* oxidase (CcO) is an important biomarker for energy-demanding tissues like the muscle [11,12,13,14]. CcO is the last electron acceptor in the ETC, reduces oxygen to water, and its activity serves as a key parameter of muscle oxidative capacity and health, ideally in conjunction with cellular energy levels [15,16]. Basal CcO activity, however, can increase with endurance exercise interventions, whereas interventions such as detraining result in a significant reduction in the skeletal muscle [17].

Studies indicate that additional mitochondrial markers have been linked to aging-induced skeletal muscle dysfunction. For example, adult mice null for Parkin, an important protein involved in mitochondrial quality control, exhibit impaired skeletal muscle mitochondrial bioenergetics similar to those seen with aging [18,19,20]. In contrast, Parkin overexpression is associated with reducing muscle atrophy in a rodent model of sepsis [21]. Mitochondrial transcription factor A (TFAM) is key in regulating the replication, transcription, and maintenance of mitochondrial DNA, and overexpression of TFAM attenuates hindlimb atrophy in a rodent model of immobilization [22]. BNIP3 (BCL2/adenovirus E1B interacting protein 3) has been implicated in contributing to the overall function of the mitochondria via mitophagy [23]. Indeed, studies show that the knockdown of BNIP3 in myotubes results in altered mitophagy which, in turn, disrupts mitochondrial homeostasis [23]. Another regulator of mitophagy is Drp1 (dynamin-related protein 1) which is involved in mitochondrial fission [24]. Drp1 muscle-specific deletion studies indicate that the rodent develops mitochondrial dysfunction which may, in part, contribute to muscle wasting [25]. Therefore, gaining insight into the interplay between mitochondrial dysfunction in aging skeletal muscle and sarcopenia could offer valuable information for developing interventions and identifying therapeutic targets to alleviate age-related muscle decline and promote healthy aging.

Strategies to reverse or attenuate aging-induced mitochondrial dysfunction in skeletal muscle focus on different exercise modalities (i.e., endurance and/or strength training). For example, McKenna and colleagues [26] reported that 10 weeks of a progressive resistance training improved mitochondrial function in a group of middle-aged men. Similarly, Mesquita et al. [27] reported that chronic resistance training increased protein levels of all five mitochondrial complexes in the skeletal muscle of older adults. Grevendonk et al. [28] identified a correlation between regular physical activity and improved mitochondrial function in a group of older, healthy adults. Johnson and colleagues [29] reported that 8 weeks of endurance training increased antioxidant capacity in older adults to counteract the aging-induced oxidative stress in the skeletal muscle. Recently, Hendrickse et al. [30] showed that incorporating endurance training improves mitochondrial function in older adults and, alongside this, that resistance training does not diminish the observed muscle hypertrophy linked to resistance training. Although different forms of exercise modality appear to be beneficial to attenuate aging-induced mitochondrial dysfunction it presupposes that the individual has a requisite level of mobility [31]. 

Non-exercise alternatives such as nutraceuticals or pharmacological agents to improve skeletal muscle bioenergetics act systemically and have resulted in moderate success. For example, (−)-epicatechin, a bioactive compound primarily found in cocoa, was shown to improve skeletal muscle bioenergetics either alone [32,33] or in combination with endurance training [33] in an aging rodent model. Similarly, resveratrol (found in red grapes) [34], quercetin (found in elderberries) [35], and the plant hormone abscisic acid which is also present in animals [36] have been shown to improve skeletal muscle bioenergetics as well as attenuate atrophy due to aging [34] or disease [35]. Moreover, pharmacological mimetics of exercise such as AMPK and PPARδ agonists [37] or AHN (adult hippocampal neurogenesis) in combination with BDNF (brain-derived neurotrophic factor) [38] have improved skeletal muscle and cognitive function in rodent models. Nevertheless, these natural and pharmacological compounds have limitations, particularly in the duration of time (i.e., weeks or months) to induce beneficial molecular and cellular changes in skeletal muscle. Thus, the question arises: “Is there a faster, tissue targeted, and more effective approach to enhance skeletal muscle bioenergetics?”

Mitochondrial transplantation represents a novel therapy designed to enhance energy production of tissues impacted by defective mitochondria [31]. Pioneered by the McCully laboratory [39,40,41,42], this innovative approach involves transferring isolated mitochondria from either a donor to a host or from the host to itself. Initially used to attenuate the effects of ischemia–reperfusion injury in cardiac tissue [43,44] transplanted mitochondria, which are rapidly purified and remain viable and capable of respiration, are directly injected into the target tissue [39,40,41,42]. Using pediatric patients that required post-cardiotomy ECMO (extracorporeal membrane oxygenation) for severe refractory cardiogenic shock causing ischemia–reperfusion injury, Guariento et al. [45] reported significant improvements in cardiac function following autologous mitochondrial transplantation. In skeletal muscle, mitochondrial transplantation has proven effective in enhancing hindlimb bioenergetics in various rodent models, including those involving ischemia–reperfusion injury [42], local chemically induced tissue damage [46], and inherent skeletal muscle dysfunction developed over 40 generations in rodents [31]. Consequently, mitochondrial transplantation circumvents the limitations of both exercise and non-exercise interventions by directly delivering isolated mitochondria into the target tissue.

To date, no studies have used mitochondrial transplantation as an intervention to attenuate aging-induced skeletal muscle mitochondrial dysfunction. The purpose of this investigation, therefore, was to determine whether mitochondrial transplantation can enhance skeletal muscle bioenergetics in an aging rodent model. We hypothesized that mitochondrial transplantation would result in sustained skeletal muscle bioenergetics leading to improved functional capacity and found that mitochondrial bioenergetics are significantly improved.

## 2. Materials and Methods

### 2.1. Overall Experimental Design

This was a between-subjects study in which female mice that were 24 months old were randomized into one of two groups (placebo or mitochondrial transplantation). The mitochondrial transplantation involved intramuscular injection of isolated mitochondria into the hindlimb muscles (quadriceps femoris, soleus, and plantaris) of both left and right hindlimbs. All mice underwent an incremental exercise test on a rodent treadmill before the intervention, 3 weeks after the intervention, and 6 weeks after the intervention. Thereafter, all rodents were euthanized and the hindlimb muscles were harvested for various molecular and cellular analyses.

### 2.2. Animals and Housing

Mice were 18 months old, female C57BL/6J (*n* = 17; Jackson Laboratories, Inc., Bar Harbor, MN, USA) and were maintained in the university’s animal housing facility until there were 24 months old. Rodents were placed 3–4 per cage and fed a standard diet without limitations. Each rodent was randomly assigned to one of two groups (placebo or mitochondrial transplantation). Two rodents were randomly selected to be donors to isolate their hindlimb mitochondria for the transplantation. The room temperature was kept at 21 °C with 12-h light–dark cycles. All animal care and experimental procedures were approved by the Wayne State University Institutional Animal Care and Use Committee (IACUC-22-03-4436).

### 2.3. Treadmill Test

We followed the recommendations of Brossia-Root et al. [47] and were consistent with our previous studies [17,32,33,48,49]. Briefly, familiarization with the treadmill was carried out for all mice during the week prior to the incremental test. All animals ran on the treadmill (1055MSD Exer-6M, Columbus, OH, USA) at a slow speed (~5 m/min) at a 5-degree incline for approximately 5–10 min. On the test day, animals began with a warm-up at 4 m/min for 2 min, followed by an increase of 2 m/min every minute thereafter. A shock grid (0.2 mA) at the end of the treadmill belt was used to discourage the mice from stopping while the treadmill belt was moving. The test was terminated for each rodent when they were unable to consistently run on the upper two-thirds of the treadmill [17,32,33,47,48,49]. The test was repeated on three separate occasions: (1) prior to the intervention; (2) 3 weeks after the intervention; and (3) 6 weeks after the intervention.

### 2.4. Mitochondrial Transplantation Protocol

We used the same methodology to isolate mitochondria as in our previous study [31]. Briefly, two female mice (same strain and age) from the pool were randomly selected to be donor mice. The mice were euthanized (overdose, i.p. 200 mg/kg, Euthanasia III) and the quadriceps femoris muscles from both hindlimbs were quickly harvested. Thereafter, multiple 6 mm biopsy punches (Medex Suppl; Fisher Scientific, Waltham, MA, USA) were taken from the muscle and placed in separate prechilled dissociation C tubes (Miltenyi Biotec Inc., Auburn, CA, USA) with 5 mL of buffer (pH 7.2, 300 mM sucrose, 10 mM K-HEPES, and 1 mM K-EGTA). [39,40,41,42]. The C tubes were then placed in a tissue dissociator (gentleMACS™ Dissociator; Miltenyi Biotec Inc., Auburn, CA, USA) for 60 s. Afterwards, the C tubes were placed on ice and 200 µL of Subtilisin A (P5380 Millipore Sigma, St. Louis, MO, USA) was added to each tube and allowed to incubate for 10 min [40]. The tubes were then centrifuged at 750 g for 4 min and the supernatant was serially filtered through a 40 µm (Pluri-select, 43-50040-51, PluriSelect USA, El Cajon, CA, USA), 10 µm (Pluri-select, 43-500010-03, PluriSelect USA, El Cajon, CA, USA), and 5 µm cell strainer (Pluri-select, 43-50005-13, PluriSelect USA, El Cajon, CA, USA). Mitochondria were collected by transferring the filtrate to sterile 1.5 mL Eppendorf tubes and centrifuged at 9000× *g* at 4 °C for 5 min [40]. The final step required removing the supernatant and resuspending the pellets into 1 mL of the same buffer described above which resulted in 1 × 10^9^ mitochondria/mL [39,40,41,42]. Thereafter, mice received an intramuscular injection of either the placebo (i.e., vehicle) or isolated mitochondria in each of the following muscles per hindlimb: quadriceps femoris (100 µL), tibialis anterior (60 µL), and gastrocnemius complex (80 µL). The adjustments made to these volumes from our prior study [31] aimed to accommodate the atrophy observed in these older mice and to avoid tissue rupture during transplantation. The injection sequence commenced from the muscle’s proximal site and progressed through five injections to reach the muscle’s distal site [31]. This method was implemented to prevent any potential muscle injury in the localized area [31].

### 2.5. Harvesting and Preparation of Tissues

Five days after the last treadmill test (i.e., week 6 post-transplantation), all rodents were euthanized (overdose, i.p. 200 mg/kg, Euthanasia III). The hindlimbs muscles were harvested, serially cut, placed in Eppendorf tubes, and immediately flash-frozen in liquid nitrogen. Tissues were then used for various analyses described below.

### 2.6. Cytochrome c Oxidase (CcO) Activity and ATP Concentrations

To examine the effect of mitochondrial transplantation on respiration kinetics, 5 µg mitochondria isolated were used in conjunction with our standard protocol, following sonication of the mitochondria and the addition of cytochrome *c* and the reductant ascorbate as described [50]. CcO-specific activity was analyzed with a micro Clark-type oxygen electrode in a closed chamber (Oxygraph system, Hansatech, Norfolk, UK) at 25 °C. Oxygen consumption was recorded on a computer and analyzed with the OxyTrace+ software, version 1.0. CcO-specific activity was defined as consumed O_2_ (nmol)/min∙mg total protein and normalized to the placebo group. 

ATP levels were analyzed by applying the boiling method with flash-frozen quadriceps femoris tissue samples using the ATP bioluminescence assay kit HS II (Roche Applied Science, Penzberg, Germany) as described [50,51]. Data were standardized to the protein concentration, determined as described above, and normalized to the placebo group.

### 2.7. Muscle Homogenization and Mitochondrial Enrichment for Blue Native PAGE (BN-PAGE)

Quadriceps femoris (30 mg) homogenization performed using the C tubes and the gentleMACS™ tissue dissociator, MACS, Selby, South Africa) for 60 s in 2 mL mitochondrial isolation buffer (MIB: 200 mM sucrose, 10 mM Tris, 1 mM EGTA, pH 7.4 adjusted with 1 M HEPES solution (Gibco, cat. No. 15630080) supplemented with PMSF (phenylmethylsulfonyl fluoride; 1 mM). The entire mitochondrial isolation procedure was performed on ice. The tissue homogenate was filtered through 40 µm strainers, then 10 µm strainers as described above, omitting the 5 µm filter. Finally, the mitochondrial fraction was collected through high speed centrifugation step (17,000× *g*, 10 min, 4 °C).

### 2.8. Mitochondrial Membrane Solubilization for BN-PAGE

The BN-PAGE protocol was adapted from [52,53]. All the following buffers and reagents steps were part of the NativePAGE™ Sample Prep Kit (Thermo Fisher Scientific, Waltham, MA, USA, cat. No. BN2008) including NativePAGE™ 4× sample buffer, NativePAGE™ 5% G-250 sample additive, and digitonin (5%). To release mitochondrial proteins embedded in the inner mitochondrial membrane, the gentle detergent digitonin was used to preserve and separate higher molecular structures including supercomplexes in their native and active form under non-denaturing conditions. As digitonin can precipitate, it is important to heat the solution at 95 °C for 5 min, gently vortex it to dissolve any precipitate, and then cool it on ice before use. Mitochondria were solubilized in NativePAGE™ 4× sample buffer supplemented with digitonin (5%) to achieve a 2% final concentration (digitonin/protein ratio (8 g/g)). The mixture was then incubated on ice for 20 min to extract the protein complexes from the inner mitochondrial membrane, followed by a centrifugation step at 4 °C and 17,000× *g* for 30 min. Next, the supernatant containing the solubilized proteins was supplemented with 0.5% G-250 sample additive. Samples (50 µg) were loaded on native 3–12% gradient gels (Invitrogen, Carlsbad, CA, USA, cat. No. BN1001BOX). Blue cathode buffer (50 mM Tricine, 15 mM Bis-Tris, 0.02% G-250 dye, pH 7.0) and anode buffer (50 mM, Bis-Tris, pH 7.0) were prechilled before use. The electrophoresis was carried out at 4 °C at 150 V until the desired separation was achieved.

### 2.9. Mitochondrial Native Protein In-Gel Activity Assay (IGA)

Complex I in-gel activity assay (IGA) following BN-PAGE was performed as described [53]. The native gel was incubated in 20 mL fresh complex assay buffer (2 mM TrisCl, pH 7.4, 0.1 mg/mL NADH, 2.5 mg/mL nitrotetrazolium blue chloride (NTB)) at room temperature until violet bands appeared. 

### 2.10. Citrate Synthase Activity

Consistent with our previous studies, basal citrate synthase activity was measured in the plantaris muscle of animals for each group using the method of Srere [54]. Samples were analyzed in a Beckman DU 730 spectrophotometer (Beckman, Fullerton, CA, USA) at 412 nm. All samples were tested in triplicate and measured at room temperature.

### 2.11. Western Blot

Our western blot analyses were consistent with our previous publications [17,31,32]. Thirty milligrams of the soleus and plantaris muscles were separately homogenized (Bead Ruptor 4, Omni International, Kennesaw, GA, USA) with chilled RIPA buffer (R0278, Sigma-Aldrich, Burlington, MA, USA) and protease and phosphatase inhibitor cocktails (PhosSTOP Phosphatase and Complete Protease Inhibitor Cocktail, Roche Applied Science, Indianapolis, IN, USA). The total protein concentration was determined by the bicinchoninic acid method (BCA protein assay kit, Bio-Rad, Hercules, CA, USA).

Protein samples (40 µg) were incubated at 95 °C for 5 min and loaded onto 12% TGX pre-cast gels (Bio-Rad) and ran for 1 h at 190 V. Odyssey blocking buffer (LI-COR Biosciences, Lincoln, NE, USA) was used on the membranes after semi-dry blotting (9 V, 50 min, Bio-Rad, Hercules, CA, USA) for 1 h at room temperature. Thereafter, the primary antibody (Table 1) was used for overnight incubation at 4 °C with gentle shaking. On day two, the membranes were washed 4 times every 5 min in Tris buffered saline with Tween 20 (TBST) wash solution. Then, the membranes were incubated with secondary antibodies (Table 1) for 1 h with the same washing procedure. The Odyssey infrared imaging system was used to quantify the blots (LI-COR Biosciences, Lincoln, NE, USA). The loading control for target proteins were normalized to α-tubulin. The data are reported as normalized to the placebo group [17,31,32]. Original western blots can be found at Supplementary Materials.

### 2.12. Epigenetic Analyses

DNA was extracted from the quadriceps femoris muscle samples using the Qiagen AllPrep Kit on a Qiagen Qiacube automated machine (Qiagen, Hilden, Germany). The DNA was quantified on a Qubit fluorometer, and 500 ng of DNA was bisulfite-converted using the Qiagen Epitect Bisulfite Kit. Pre-designed methylation primers were ordered from Qiagen for the following genes: Nfe2L2, Ppargc1b, SIRT3, and TFAM. The primer specifications and locations can be found on the Qiagen website; however, they are generally within CpG islands of each gene and cover between 3 and 10 CpG sites per primer set. Bisulfite PCR was then performed with 20 ng of bisulfite converted DNA and PCR amplicon was checked on a Qiagen QIAxcel Advanced System. Each gene’s amplicon was sequenced according to manufacturer specifications on a Qiagen Automated Q48 Pyrosequencer which computes a ratio based on the signal generated by the incorporation of a methylated cytosine versus an unmethylated cytosine (a thymine due to bisulfite conversion). The pyrosequencing software transforms these ratios, accounting for background signal, into methylation values which are a percentage of methylation from 0 to 100%. These methylation values were then extracted for further statistical analysis.

### 2.13. Statistical Analyses

All data in the present study are presented as mean ± SEM (standard error of the mean) values. To determine changes in exercise tolerance we performed separate 2 [group: placebo or mitochondrial transplantation] × 3 [time: pre-intervention, 3 weeks, and 6 weeks post-intervention] mixed factorial ANOVAs for the various exercise indices. For all other comparisons, an independent samples *t*-test was used to compare mean differences between the two groups. All statistical analyses were performed using Statistical Package for the Social Sciences software (v. 28.0, IBM SPSS, Armonk, NY, USA) with an alpha level set at *p* ≤ 0.05.

## 3. Results

### 3.1. Anthropometrics

In this study, 24-month-old female mice were subjected to skeletal muscle mitochondrial transplantation. Rodent body mass was measured on several occasions throughout the experiment. In addition, once rodents were euthanized, hindlimb muscles were harvested and weighed. As shown in Table 2, the mixed factorial ANOVA revealed no significant group X time interaction [F(2,28) = 0.380; *p* = 0.688] for body mass. In addition, there were no significant main effects for body mass [F(2,28) = 1.826; *p* = 0.180] or group [F(1,14) = 0.247; *p* = 0.627]. The separate independent samples *t*-tests revealed no significant mean differences (*p*-values ranged from 0.221 to 0.962) for absolute and relative muscle mass between the two groups when animals were euthanized 6 weeks after mitochondrial transplantation (Table 2).

### 3.2. Exercise Tolerance

**Figure 1:**
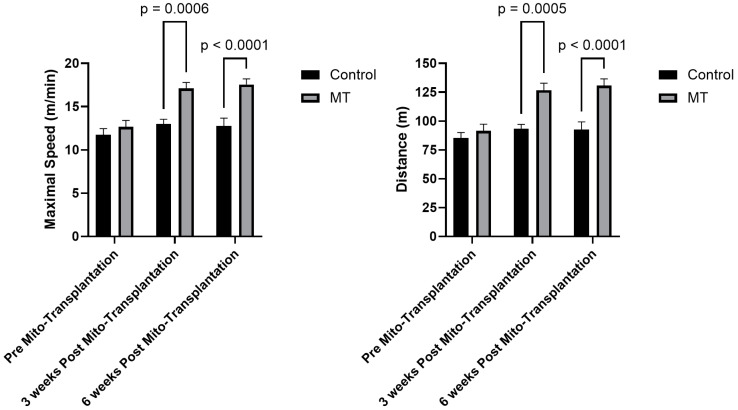
Results of the incremental treadmill test, prior to, 3 weeks after, and 6 Weeks after mitochondrial transplantation (mean ± SEM; *n* = 7 to 8 mice per group). Note: MT: mitochondrial transplantation.

Prior to the formal analyses for maximal speed, we tested for the sphericity assumption. The results indicated a non-significant relationship (*p* = 0.285), indicating that the assumption was met. The 2 × 3 mixed factorial ANOVA revealed a significant group-by-time interaction [F(2,28) = 9.39; *p* < 0.001] for the maximal speed achieved. There was also a significant main effect for speed [F(2,28) = 22.85; *p* < 0.001] and group [F(1,14) = 20.13; *p* < 0.001]; however, due to the significant interaction, these main effects were not discussed. After the significant interaction, the post hoc LSD (least significant difference) was performed to determine significant differences between the groups at each time point. As shown in Figure 1, there were no significant mean differences between groups prior to the intervention, however, 3 weeks after the intervention the group receiving the mitochondrial transplantation achieved a higher maximal speed (~45%) relative to the placebo group which was maintained at 6 weeks post-transplantation (Figure 1). 

Prior to the formal analyses for total distance, we tested for the sphericity assumption. The results indicated a non-significant relationship (*p* = 0.274) indicating that the assumption was met. The 2 × 3 mixed factorial ANOVA revealed a significant group by time interaction [F(2,28) = 10.14; *p* < 0.001] for total distance ran. There was also a significant main effect for speed [F(2,28) = 21.62; *p* < 0.001] and group [F(1,14) = 20.62; *p* < 0.001]; however, due to the significant interaction, these main effects were not discussed. After the significant interaction, the post hoc LSD was performed to determine significant differences between the groups at each time point (Figure 1). There were no significant differences between the groups prior to the intervention; however, 3 weeks after the intervention the group receiving the mitochondrial transplantation ran for longer (~48%) relative to the placebo group which was maintained at 6 weeks post-transplantation (Figure 1).

### 3.3. CcO and Complex I Activity, [ATP], and Supercomplex Composition

**Figure 2:**
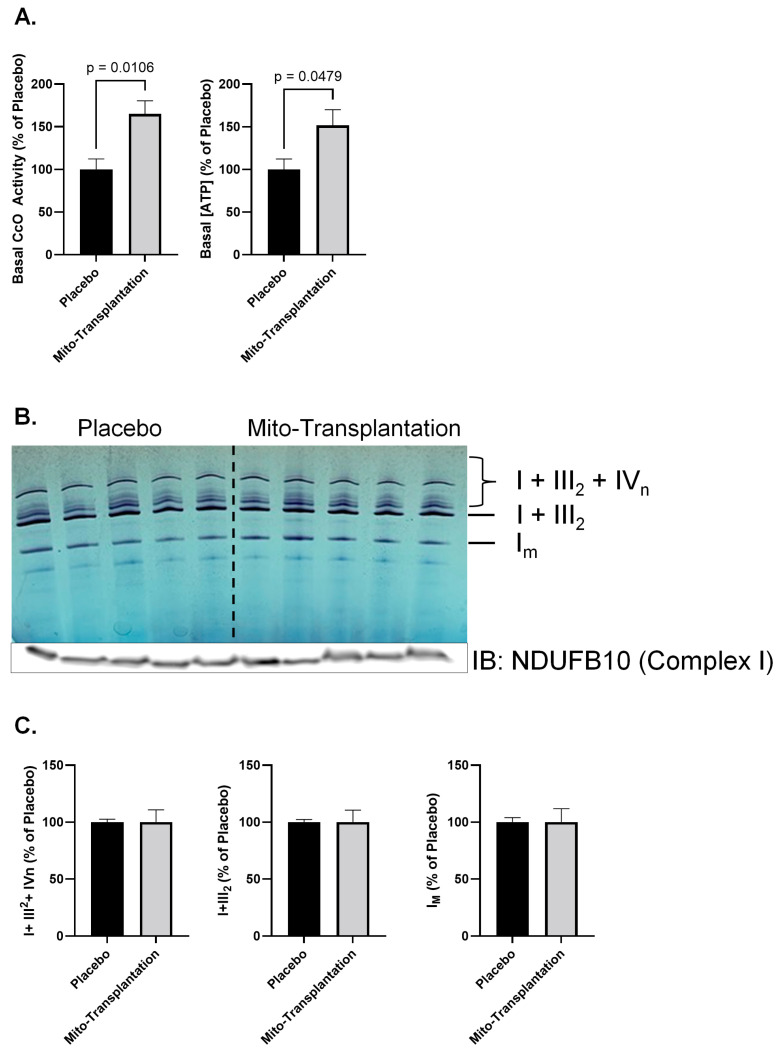
Basal cytochrome *c* oxidase activity and [ATP] in the quadriceps femoris muscle (**A**). High-resolution complex I-related in-gel activity assay reveals no significant changes in supercomplex composition in aged mouse quadriceps muscle following mitochondrial injection. The gel was incubated with a complex I substrate, and the violet bands indicate complex I in-gel activity (**B**). (**C**) is the quantitative representation of supercomplex from panel (**B**). SC denotes supercomplex, while I denotes monomeric complex I. Each lane represents an individual sample from a different mouse (mean ± SEM).

To investigate the impact of mitochondrial transplantation on mitochondrial bioenergetic function, we first assessed the activity of CcO, which is considered the rate-limiting step in the electron transport chain under physiological conditions, along with ATP levels. As shown in Figure 2, our analysis indicated a significant mean difference [*t* (8) = 3.313; *p* = 0.0106] in basal CcO activity for the quadriceps femoris muscles, which was increased by ~65% 6 weeks following mitochondrial transplantation. In addition, we found a similar pattern of responses [*t* (11) = 2.226; *p* = 0.0479] for basal [ATP], which was increased by ~51% in the same muscle Figure 2.

We also utilized Blue Native Polyacrylamide Gel Electrophoresis (BN-PAGE) analysis, a technique for studying electron transport chain supercomplex (SCs) assemblies, which are comprised of monomeric Complex I, dimeric Complex III, and various stoichiometries of Complex IV. BN-PAGE revealed no significant (*p >* 0.05) SCs composition changes following mitochondrial transplantation, nor significant changes in complex I-related in-gel activity, further highlighting the role of CcO as a key determinant for improved bioenergetic function post-transplantation.

### 3.4. Epigenetic Response

The independent samples *t*-tests indicated no significant mean differences for global DNA methylation for mitochondria markers NFe2L2 [*t* (13) = 0.952; *p* = 0.359], Ppargc1b [*t* (13) = 0.019; *p* = 0.986], SIRT3 [*t* (13) = 1.90; *p* = 0.080], and TFAM [*t* (13) = 1.074; *p* = 0.302]. 

### 3.5. Basal Citrate Synthase Activity in the Plantaris Muscle

We normalized the citrate synthase activity to the placebo group (% placebo) prior to the formal analyses. The independent samples *t*-test revealed a significant mean difference for basal citrate synthase in the plantaris muscle [*t* (10) = 2.721; *p* = 0.0215] between the placebo (mean ± SEM; 100 ± 11%) and mitochondrial transplantation (137 ± 7%) groups. 

### 3.6. Western Blots for Mitochondrial Protein Expression

**Figure 3:**
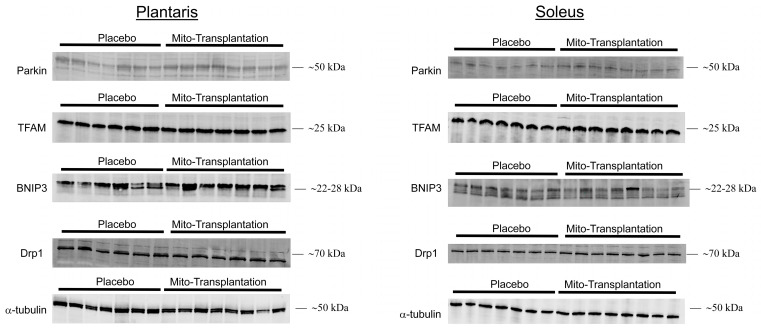

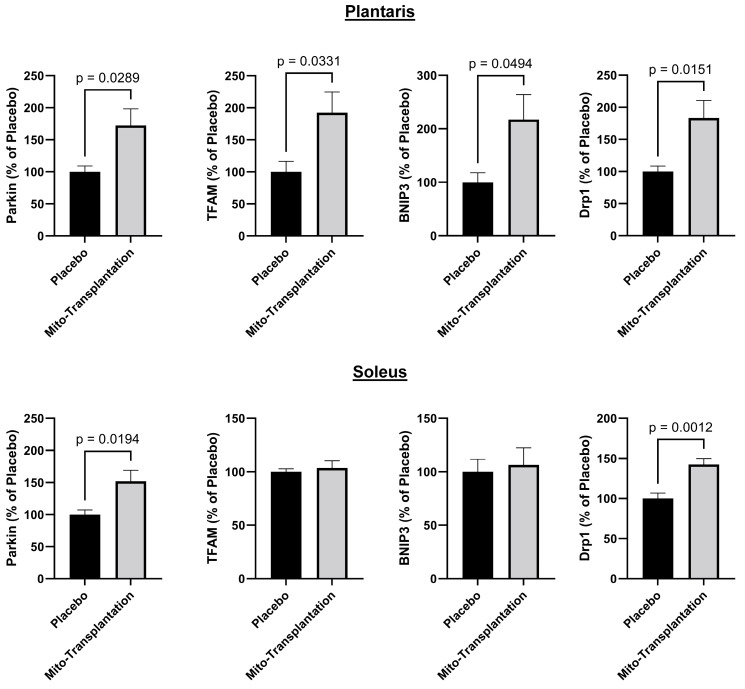
Representative western blots and quantification for key mitochondrial markers for the soleus and plantaris muscles. Loading control for target proteins were normalized to α-tubulin (mean ± SEM; *n* = 5–8 per group).

For the plantaris muscle (Figure 3, left panel), separate independent samples *t*-tests indicated significant mean differences between the placebo and mitochondrial transplantation groups for the protein Parkin [*t* (13) = 2.455; *p* = 0.0289], TFAM [*t* (11) = 2.436; *p* = 0.0331], BNIP3 [*t* (11) = 2.207; *p* = 0.0494], and Drp1 [*t* (10) = 2.973; *p* = 0.0151]. All four protein markers were increased between 1.7- and 2-fold. However, for the soleus muscle (Figure 3, right panel), the separate independent samples *t*-tests indicated significant mean differences for Parkin [*t* (13) = 2.665; *p* = 0.0194] and Drp1 [*t* (13) = 4.140; *p* = 0.0012] between the two groups.

## 4. Discussion

The present investigation revealed unique outcomes, showcasing that mitochondrial transplantation improved skeletal muscle bioenergetics in female mice that were 24 months old. Indeed, we found increased CcO activity and, in parallel, significantly improved energy levels. This enhancement led to an improved exercise tolerance compared to the placebo group, persisting for six weeks after mitochondrial transplantation. The salient feature of this study, however, resides in the fact that the isolated mitochondria originated from donor mice of same sex, strain, and age, rather than from younger rodent donors. This study marks the initial evidence showing how mitochondrial transplantation can promote skeletal muscle bioenergetics in an aging rodent model.

It may seem reasonable to assume that isolating mitochondria from a younger donor would be advantageous compared to isolating mitochondria from a diseased or older donor. There is, however, research indicating the effectiveness of using isolated mitochondria from a donor with similar characteristics to the recipient. For example, Doulamis and colleagues [55] isolated mitochondria from diabetic and healthy rats. Thereafter, these isolated mitochondria were injected into the cardiac muscle of rodents who underwent ischemia–reperfusion injury. The authors found comparable reductions in infarct size when isolated mitochondria were derived from diabetic or healthy rats [55]. The investigators, however, found that proteomic alterations in the metabolism profile of the cardiac muscle were more obvious in rodents that received isolated mitochondria from non-diabetic rodents rather than from diabetic rodents [55]. Recently, Arroum et al. [31] examined the effects of mitochondrial transplantation in the skeletal muscle of rats that were bred for innate low running capacity (LCR) over 40 generations. These rodents exhibited mitochondrial dysfunction in the hindlimb muscles akin to a severely deconditioned individual [56,57,58]. Interestingly, the investigators used isolated mitochondria from a donor LCR rodent and then transplanted the mitochondria into recipient LCR rodents of the same sex and age. Arroum and colleagues [31] reported improvements in hindlimb muscle bioenergetics which included significant increases in basal CcO activity and [ATP] as well as increased protein expression of various markers of mitochondria biogenesis. 

In the present study, we used two donor female mice that were 24 months old (equivalent to 70-year-old humans [59]) from the group of mice used for these experiments. The mitochondria were isolated from the quadriceps femoris muscles of these two donors and then transplanted into the hindlimb muscles of recipient mice. Six weeks after mitochondria transplantation we found that basal CcO activity and [ATP] in the quadricep femoris muscles of mice receiving mitochondrial transplantation was ~65% and ~51% greater than mice receiving the placebo, respectively. In addition, we observed a ~37% increase in basal citrate synthase activity in the plantaris muscle in mice receiving mitochondrial transplantation. These increases in basal CcO and citrate synthase activities as well as [ATP] in the skeletal muscle are lower than the values reported in rodent (ranging from ~80% to ~105%) [17,60] and human (ranging from ~30% to ~60%) [61,62] studies utilizing endurance training, and future studies should explore the potential additive or synergistic effect of mitochondrial transplantation in combination with endurance training. 

In addition to these changes that were observed, we also detected significant increases in protein expression of Parkin and TFAM in the plantaris muscles of mice receiving mitochondrial transplantation (Figure 3). Our results are consistent with those of Arroum et al. [31] who reported significant increases in Parkin and TFAM protein expression in the plantaris of rats 3 weeks after receiving mitochondrial transplantation. Similarly, we and Arroum et al. [31] also found significant increases in Parkin protein expression in the soleus muscle but no changes in TFAM protein expression. The increased protein expression of Parkin in both glycolytic (i.e., plantaris) and oxidative (i.e., soleus) muscles may be, in part, explained by the role of Parkin in mitophagy. Indeed, in both plantaris and soleus muscles, we also found that Dpr1 protein expression was significantly higher in mice receiving mitochondrial transplantation (Figure 3). Muscle-specific Drp1 knockout mice have mitochondrial dysfunction with a concomitant loss in sustaining mitochondrial quality control [25]. Moreover, King and colleagues [63] reported that the loss of Drp1 contributed to disruptions in mitochondrial metabolism with specific impairments associated to glucose uptake and fatty acid oxidation. Dulac et al. [64] reported that the knockdown of Drp1 or overexpression of Drp1 in 18-month-old mice resulted in negative outcomes in the skeletal muscle. The authors concluded that maintaining Drp1 content within a narrow physiological range is optimal to sustain mitochondrial integrity [64]. It should be noted, however, that the phosphorylated state of Drp1 may adversely influence mitochondrial function in the skeletal muscle of cancer-induced cachectic patients [65]. We also found that BNIP3 was significantly increased in the plantaris muscle, but not in the soleus muscle. In cardiac myocytes, Lee et al. [66] reported that a Drp1 inhibitor prevented BNIP3 from facilitating the relocation of Parkin to the mitochondria. Wang and colleagues [67] reported a significant increase in BNIP3 protein expression in the hindlimb muscle of rats after three days of remobilization subsequent to a two-week immobilization period. Moreover, it has been suggested that BNIP3 facilitates mitophagy in order to maintain healthy mitochondria of the muscle during aging [68,69]. Taken together, our data suggest that mitochondrial transplantation, in an aging rodent model, may provide the optimal dose of mitochondria to increase enzymatic activity and sustain quality control without adversely affecting mitochondrial function.

The functional manifestations of the improved bioenergetics of mice receiving mitochondrial transplantation, in the current study, resides in the improved exercise tolerance test that was performed 3 and 6 weeks after the intervention. The incremental treadmill protocol test used in the current study maintains a constant 5% grade, whereas the speed of the belt is increased 2 m/min after the warm-up phase. This protocol was purposely designed to test rodents across various interventions which either increased exercise tolerance such as endurance training [17] or supplementation [33], but also for conditions where exercise tolerance was compromised by genetic deletion [51]. Nevertheless, in the present investigation, we observed that exercise capacity increased 3 weeks after mitochondrial transplantation relative to the placebo group. Importantly, these improvements in functional capacity were not transient and sustained 6 weeks after mitochondrial transplantation. Mitochondrial protein turnover is both protein- and tissue-specific. For mouse heart muscle tissue, in one of two tissues studied that is more similar to skeletal muscle than liver (the second studied tissue), the protein median half-life was 17.2 days [70]. If protein turnover is similar in heart and skeletal muscles, one can propose that the newly introduced mitochondria remain integrated and functional as suggested by sustained exercise performance at 3 and 6 weeks post-transplantation. Further investigation, however, is needed to determine whether the functional improvements persist beyond 6 weeks or whether there is a return to baseline levels at later time points.

**Figure 4:**
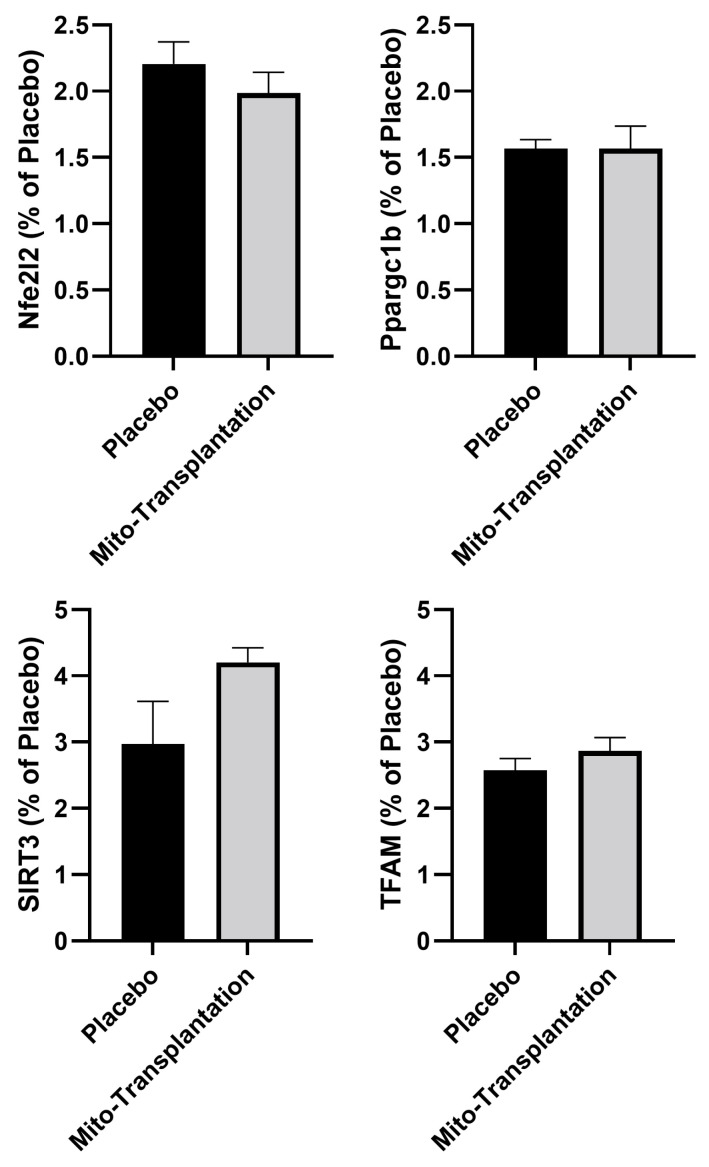
Epigenetic results for the quadriceps femoris muscles for NFe2L2, PPAGC-1β, SIRT3, and TFAM. No significant (*p* > 0.05) mean differences between groups (mean ± SEM; *n* = 7 to 8 mice per group).

In the current study, we did not observe any statistically significant changes in DNA methylation (Figure 4). This could be due to numerous factors. First, DNA methylation varies depending on the tissue of interest. Moreover, in skeletal muscle there is also the distinction between muscles that are glycolytic, oxidative, or a combination of both fiber types [71]. In the present study, we selected the quadriceps femoris muscles which have a combination of glycolytic and oxidative fibers. Second, our approach for DNA methylation was a candidate gene approach focusing on a select number of genes associated with mitochondria function. Furthermore, there are many CpG islands associated with a single gene of interest, and, therefore, it is possible that other regions within the same gene could be better correlated to gene control manifesting in changes in protein expression. Third, another potential consideration is the timing of the mitochondrial transplantation relative to when the rodents were euthanized and tissues harvested. In the present study, the duration between mitochondrial transplantation and tissue harvesting was 6 weeks. Thus, it is conceivable that DNA methylation changes occur rapidly following mitochondrial transplantation leading to sustained downstream changes in protein expression while the DNA methylation signatures return to baseline later. This premise is supported by studies that have identified such an effect on DNA methylation [72,73,74]. Lastly, it has been suggested that aging can impact the epigenetic response via damage to the DNA [75,76,77]. Thus, the 24-month age of our mice might be linked to potential epigenetic dysfunctions that were not overcome by mitochondrial transplantation.

## 5. Conclusions

In summary, the findings of the present study indicate that a single mitochondrial transplantation treatment into the hindlimbs of aged rodents leads to enhanced mitochondrial function 6 weeks post-transplantation compared to the placebo group. It is worth noting that the transplanted mitochondria were harvested from donors of the same age (24 months old) rather than from younger donors. Furthermore, the improvements observed in the tissue of rodents that received the mitochondrial transplantation were predominantly from glycolytic or mixed muscle fibers. Interestingly, there were observed protein expressions of mitochondrial markers associated with mitophagy irrespective of muscle fiber type. The enhancements in skeletal muscle bioenergetics translated to improved exercise tolerance. Future studies, however, are needed to ascertain whether the sustained enhancements in mitochondria function are long-lasting or eventually revert to pre-transplantation baseline levels.

## Figures and Tables

**Table 1:** Primary and secondary antibodies used for western blots.

Antibody	Company	Product ID	Lot No.	Dilution
Parkin	Cell Signaling Technology (Danvers, MA, USA)	2132S	4	1:1000
TFAM	Abcam (Cambridge, UK)	Ab252432	GR3455740-1	1:1000
Anti-BNIP3	Abcam	Ab109362	GR3284300-7	1:1000
Drp1	Abcam	Ab184247	GR3369203-23	1:1000
α-Tubulin	Abcam	Ab7291	1009714-1	1:1000
IRDye 800CW	LI-COR (Lincoln, NE, USA)	925-32211	D11103-01	1:20,000
IRDye 680RD	LI-COR	925-68070	D10901-11	1:20,000

**Table 2:** Anthropometrics of the mice in each group (mean ± SEM).

	Placebo			Mitochondria Transplantation		
	Pre-Injection	3 Weeks Post-Injection	6 Weeks Post-Injection	Pre-Injection	3 Weeks Post-Injection	6 Weeks Post-Injection
Body mass (g)	33.7 ± 1.6	32.5 ± 1.2	33.0 ± 1.2	34.2 ± 1.7	33.5 ± 1.2	34.3 ± 1.2
Soleus (mg)	-	-	8.1 ± 0.8	-	-	7.4 ± 0.5
Soleus/body mass (mg/g)	-	-	0.25 ± 0.03	-	-	0.22 ± 0.02
Plantaris (mg)	-	-	13.8 ± 0.8	-	-	12.6 ± 0.7
Plantaris/body mass (mg/g)	-	-	0.42 ± 0.04	-	-	0.37 ± 0.02
Quadriceps femoris (mg)	-	-	141.4 ± 3.2	-	-	151.0 ± 6.8
Quadriceps femoris/body mass (mg/g)	-	-	4.32 ± 0.53	-	-	4.43 ± 0.68

Note: No statistically significant (*p* > 0.05) mean differences between groups and time points for body mass and muscle weights.

## Data Availability

All data from this study are available from the authors upon reasonable request.

## Supplementary Materials

The following supporting information can be downloaded at: https://www.mdpi.com/article/10.3390/biom14040493/s1.
