# Supplementary figures and images for: Mitochondrial Transplantation’s Role in Rodent Skeletal Muscle Bioenergetics: Recharging the Engine of Aging
Source: Biomolecules. 2024 Apr 18;14(4):493. doi: 10.3390/biom14040493 (PMC11048484; doi:10.3390/biom14040493)

## Supplemental File

### Western Blots

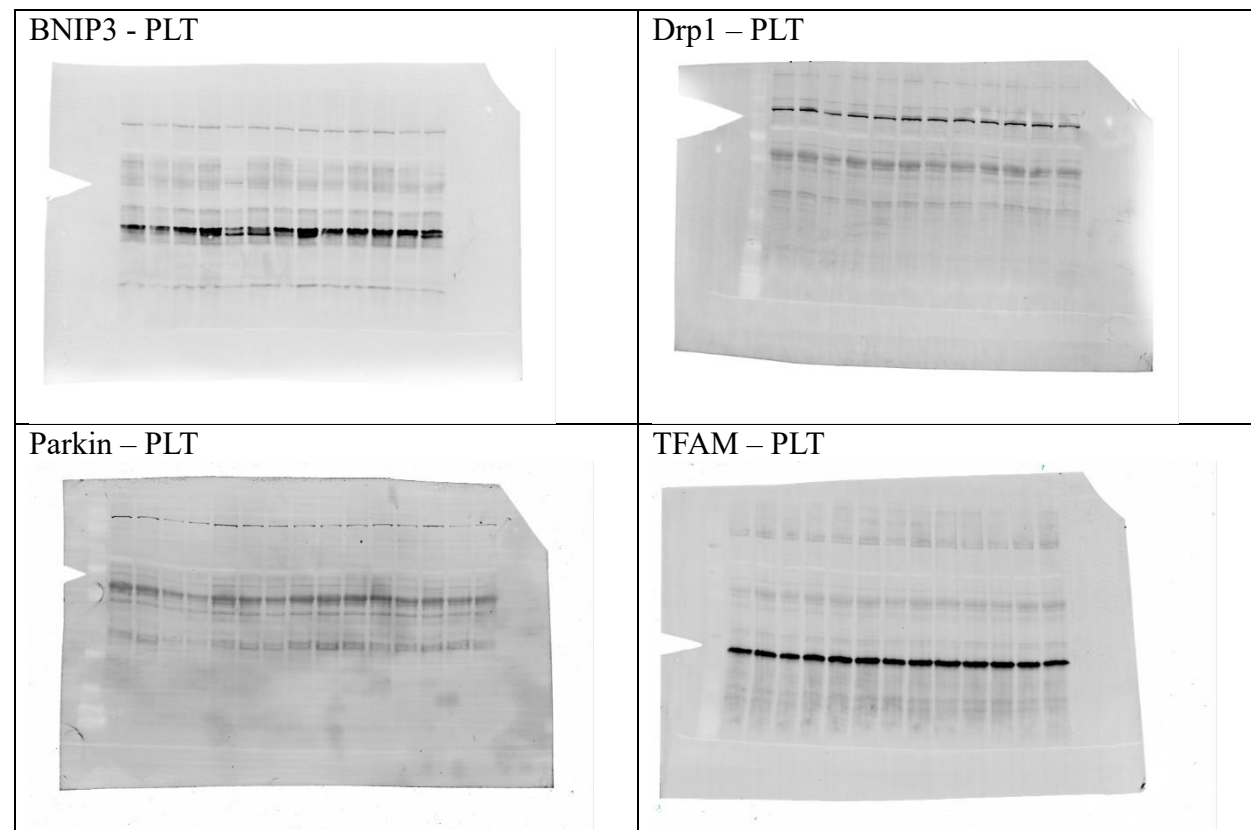

BNIP3 – SOL

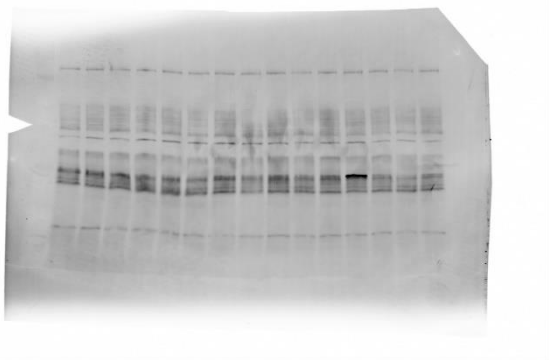

Drp1 – SOL

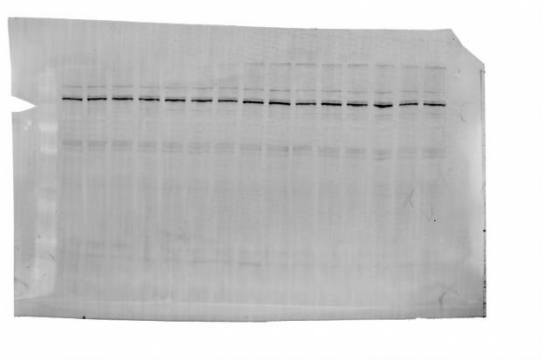

Parkin – SOL

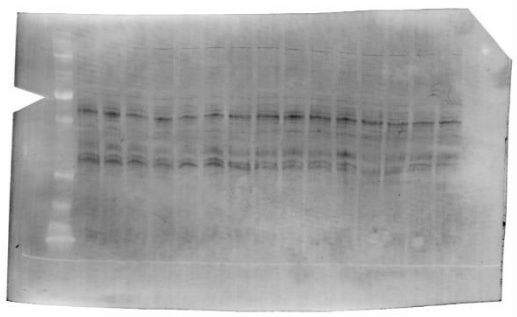

TFAM – SOL

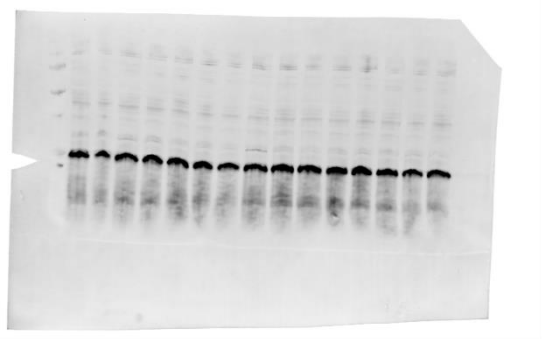

Supplement: Supplementary file 1 [file biomolecules-14-00493-s001.zip › biomolecules-2901508-supplementary.pdf]
